# Connecting the Airways: Current Trends in United Airway Diseases

**DOI:** 10.3390/jpm16010021

**Published:** 2026-01-04

**Authors:** Benedetta Bondi, Martina Buscema, Federico Di Marco, Carlo Conti, Andrea Caviglia, Lorenzo Fucci, Anna Maria Riccio, Marcello Mincarini, Martina Ottoni, Fulvio Braido, Rikki Frank Canevari, Diego Bagnasco

**Affiliations:** 1 Respiratory and Allergy Clinic, IRCCS Ospedale Policlinico San Martino, 16132 Genoa, Italy; 2 Department of Internal Medicine (DIMI), University of Genoa, 16132 Genoa, Italy; 3 ENT Department, IRCCS Policlinico San Martino, University of Genoa, 16132 Genoa, Italy; 4 Allergy and Clinical Immunology, Medicine and Surgery Department, University of Parma, 43121 Parma, Italy

**Keywords:** united airway disease (UAD), type 2 inflammation, biologic therapy

## Abstract

The concept of united airway disease (UAD) highlights the bidirectional relationship between inflammatory disorders of the upper airways—such as allergic rhinitis and chronic rhinosinusitis with or without nasal polyps (CRSwNP/CRSsNP)—and lower airway diseases, most notably asthma. This paradigm is supported by epidemiological, embryological, and immunological evidence demonstrating that airway inflammation represents a single, interconnected process rather than isolated compartmental pathology. Central to many UAD phenotypes is type 2 (T2) inflammation, driven by cytokines including IL-4, IL-5, and IL-13, and mediated by effector cells such as eosinophils and group 2 innate lymphoid cells (ILC2s). Epithelial barrier dysfunction often serves as the initiating trigger for this shared inflammatory cascade by production of TSLP, IL-25 and IL-33. Optimal diagnosis and management of UAD require an integrated, multidisciplinary framework. Clinical evaluation remains essential for patient characterization but must be complemented by pheno-endotypic assessment using imaging (CT), allergy testing, biomarker profiling (FeNO, blood eosinophils, IgE), and pulmonary function testing (spirometry, impulse oscillometry). Therapeutic strategies are layered, targeting both symptom control and inflammation across airway compartments. Standard approaches include intranasal and inhaled corticosteroids as well as saline irrigations, while severe T2-high disease increasingly benefits from biologic therapies (anti-IL-5/IL-5R, anti-IL-4R, anti-TSLP), which reduce dependence on systemic corticosteroids and surgical interventions such as endoscopic sinus surgery (ESS). Emerging precision-medicine models, particularly the “treatable traits” approach, further underscore the need to view the airway as a unified system. Collectively, these insights reinforce the clinical imperative of addressing upper and lower airway disease as a continuum, ensuring that inflammation in one district is neither overlooked nor treated in isolation.

## 1. Introduction

The concept of united airway disease (UAD) well describes the common nature of inflammatory conditions affecting both the upper airways—such as rhinitis and chronic rhinosinusitis, with (CRSwNP) or without nasal polyps (CRSsNP)—and the lower airways, most notably asthma [1]. The unified airway hypothesis postulates that these diseases are not isolated entities, but rather manifestations of a single, interconnected inflammatory process [2,3,4,5], with a high prevalence of coexistence between upper and lower airway disorders [6].

The upper and lower airways, though anatomically distinct, are in direct continuity and share fundamental histological and functional characteristics, including epithelial lining, mucociliary clearance, and immune cell infiltration. Both compartments are exposed to environmental allergens, infectious agents, and pollutants, which initiate inflammatory responses through overlapping immunological cascades [7]. Type 2 (T2) inflammation, mediated by cytokines such as IL-4, IL-5, and IL-13, and effector cells including eosinophils and ILC2s, represents a central pathogenic mechanism across many UAD phenotypes. Damage to the epithelial barrier further amplifies this shared inflammatory pathway, reinforcing the concept of a unified airway.

Beyond pathophysiology, UAD carries a substantial epidemiological burden. Allergic rhinitis affects up to 30–40% of the global population, while asthma impacts more than 300 million individuals worldwide, with frequent coexistence leading to more severe disease trajectories, impaired quality of life, and increased healthcare utilization. Patients with concomitant rhinitis and asthma experience higher rates of exacerbations, hospitalizations, and medication use compared to those with isolated disease. The economic impact is considerable, encompassing direct costs from pharmacological and surgical interventions as well as indirect costs related to absenteeism and reduced productivity. These data underscore the importance of recognizing UAD as a major public health issue, warranting integrated strategies for prevention, diagnosis, and treatment.

Recognition of UAD has important clinical implications. It challenges the traditional compartmentalized approach to airway disease management, emphasizing the need for integrated diagnostic and therapeutic strategies. Evidence suggests that treating asthma or rhinitis in isolation may fail to achieve optimal patient outcomes, as persistent inflammation in one district can perpetuate disease activity in the other. Consequently, multidisciplinary collaboration among pulmonologists, allergists, and otolaryngologists is increasingly advocated to ensure comprehensive care.

This review aims to synthesize current knowledge on UAD, with particular attention to pathophysiological mechanisms, diagnostic strategies—including biomarker profiling and imaging—therapeutic advancements such as biologic agents, and future directions in precision medicine. By framing airway disease as a continuum, the UAD paradigm provides a more holistic understanding of respiratory health and opens new avenues for improving patient outcomes.

## 2. Epidemiology and Clinical Burden

UAD is a highly prevalent condition, with prevalence estimates ranging from 10% to 40% [3,5,8]. In children with asthma, allergic rhinitis is present in 60–70% of cases, while in adults the prevalence decreases to 50–70%; among individuals with allergic rhinitis, asthma develops in 20–40% of children and in 15–30% of adults [5].

Epidemiological studies confirm that the deep associations between upper and lower airways are implicated in asthma control, lung function, leading to an increased bronchial hyperreactivity and to a reduction in function regardless of smoking status [9]. Furthermore, other studies demonstrate that the prevalence of allergic rhinitis and CRS is growing, particularly in the pediatric population, with rates of allergic rhinitis increasing from 8.4% to almost 20% in the last decade. Therefore, it can be deduced from here that every patient with rhinitis/rhinosinusitis should also be evaluated for the presence of lung diseases (and vice versa) [3,10,11,12].

This frequent coexistence is found principally in severe disease phenotypes. UAD encompasses a spectrum of diseases, including the well-known CRS, nasal polyposis, and COPD, but also the less considered bronchiectasis, all of which contribute to a significant disease burden: the presence of one airway disease often predicts or otherwise precedes the development of another, fueling the paradigm “one airway, one disease” [4,13].

Just the latter, recent studies show that the concomitant presence of upper airway diseases in patients with bronchiectasis, due to asthma, COPD, but also “pure” bronchiectasis syndrome, is not only common but also associated with an earlier worsening of the disease, a longer duration, and a higher frequency of exacerbations [14,15].

Shared pathogenesis includes eosinophilic or neutrophilic inflammation, mucociliary clearance defects, and allergic factors: allergic sensitization and impaired mucosal defense are important contributors in the pathogenesis of UAD. A recent Mendelian randomization study suggests that pediatric asthma may be a causal risk factor for the development of bronchiectasis and chronic rhinitis, but there is still no certain evidence regarding chronic sinusitis [16].

About the deep link between asthma and rhinosinusitis, literature evidence reveals that patients with upper respiratory disease alone, regardless of the presence of an atopic status, are more likely to develop asthma in their life and vice versa [17].

## 3. Mechanisms, Endotypes, and Phenotypes

The unified airway hypothesis is supported by epidemiological, histoembryological, immunological, and molecular evidence, which emphasizes the functional and pathophysiological unity of the upper and lower airways.

Epidemiologically, about 80% of asthmatic patients suffer from rhinitis, while 10–40% of patients with rhinitis develop asthma [18]. CRS is also linked to difficult-to-control asthma, frequent exacerbations, and poorer respiratory function and, if correctly treated, can lead to improved outcomes for bronchial disease as well [19,20,21,22]. These epidemiological data, another time, emphasize how closely connected the airways are and that pathological entities currently considered distinct may represent an immunological–inflammatory continuum involving the entire respiratory tree [2,23,24].

The continuity of the upper and lower airways originates at the embryological level, as both derive from the anterior intestine and closely connected embryonic structures, subsequently, they are prone to developing differentiations: pseudostratified mucociliary epithelium with goblet cells, the presence of mucosa-associated lymphoid tissue (MALT), epithelial barrier function, and innate and adaptive immune response patterns [2,25,26,27,28,29].

Type 2 (T2) inflammation is the pivot but not the only representation of the pathogenesis of numerous UAD phenotypes, principally characterized by eosinophilic infiltration and orchestrated by cells like T helper type 2 (Th2), cytokines such as IL-4, IL-5, and IL-13. These cytokines represent the junction between adaptive immunity and innate immunity, promoting and activating effector cells. Among these, type 2 innate lymphoid cells (ILC2) play an important role, releasing pro-inflammatory mediators in response to epithelial stimuli (mainly represented by IL-33, IL-25, and TSLP) [7], followed by the recruitment of Th2 cells and the intervention of eosinophils, the main effectors of tissue damage and maintenance of the inflammatory cascade [27,30,31].

The modulation of the abovementioned cytokines and cells plays a pivotal role in both upper and lower airway inflammation, and their therapeutic targeting has demonstrated clinical benefit across these sites [3,4,5,12,27,32,33].

**Figure 1:**
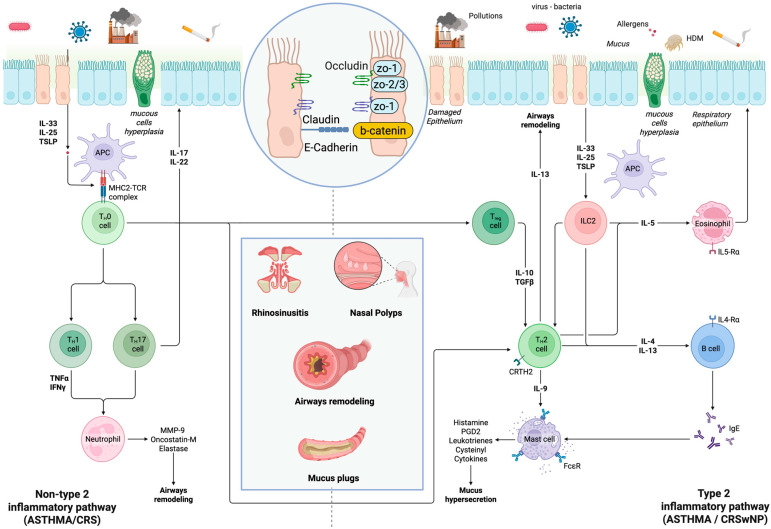
Mechanisms of inflammation in upper and lower airway diseases. Legend. *Zonulins (ZO)*, *T-helper (TH)*, *interleukin (IL)*, *prostaglandin D2 (PGD2)*, *Transforming Growth Factor Beta (TGF-β)*, *Thymic stromal lymphopoietin (TSLP)*, *Matrix Metalloproteinase-9 (MMP-9)*, *tumor necrosis factor (TNF)*, *interferon (IFN)*, *house dust mite (HDM)*, *Immunoglobulin (Ig)*, *Major Histocompatibility Complex class 2 (MHC2)*.

In asthma, as in CRSwNP, the inflammatory pathway is more often activated by a loss of epithelial barrier integrity, induced by viruses, allergens, or environmental irritants and pollutants. The loss of tight junctions, which causes a loss of cell adhesion (zonulins, claudins, occludin, ed E-cadherins), associated with defective mucociliary clearance and antibody deficiency, promotes the penetration of antigens and amplifies the immune response, generating a vicious circle that feeds inflammation and barrier damage (Figure 1). Other inflammatory pathways, including type 1 and type 3 responses, together with epithelial damage, contribute to the heterogeneity of UAD. Another aspect, although undergoing knowledge development, is the airway microbiome, including bacterial and fungal communities. It is increasingly recognized as a modulator of airway inflammation and disease expression, although its precise role in UAD pathogenesis remains under investigation [3,27,34]. This aspect is of great interest, but still too little is known to provide a clear and direct relationship between certain microorganisms and the development of disease.

MicroRNAs (miRNAs), family of small endogenous non-coding RNA molecules, have emerged as important regulators of gene expression in airway inflammation, but few have been identified that directly link upper and lower airway disease; as an example, specific miRNAs, such as miR-223-3p and miR-155, have been linked to neutrophilic inflammation and immune regulation in asthma and other airway conditions [35]. Although their clinical utility remains to be established [13].

The distinction between phenotypes (observable clinical manifestations) and endotypes (underlying biological mechanisms) is crucial in precision medicine, for understanding clinical variability and attempting to predict a response to treatment in asthma, rhinitis, and CRSwNP.

The most extensively characterized endotype is type 2 (T2) inflammation, defined by the predominance of interleukins IL-4, IL-5, and IL-13, in conjunction with eosinophilic infiltration, elevated immunoglobulin E (IgE), type 2 innate lymphoid cells (ILC2), and epithelial-derived alarmins. This immunopathological profile is typically associated with robust therapeutic responsiveness to corticosteroids—whether inhaled, intranasal, or systemic—as well as to targeted biological agents directed against key mediators, most notably monoclonal antibodies against IL-5, IL-5Rα, IL-4Rα, IL-13, or thymic stromal lymphopoietin (TSLP).

Conversely, the T2-low endotype is distinguished by a neutrophilic or paucigranulocytic inflammatory signature. It is more frequently observed in older individuals, often with increased body mass index and a history of tobacco exposure. Clinically, this phenotype remains particularly challenging, owing to the absence of reliable biomarkers and the limited efficacy of both systemic corticosteroids and currently available biologics. As such, T2-low disease represents a pressing unmet need in the field, underscoring the necessity for novel mechanistic insights and therapeutic strategies [36,37,38,39]. In addition to those described above, there are also other clinical phenotypes, such as exercise-induced asthma, aspirin-exacerbated asthma, and long-standing asthma with fixed obstruction, phenotypes that intersect with those described above, making it even more complex to identify a precise disease phenotype [39,40,41].

Regarding the upper airways, rhinitis and chronic rhinosinusitis (CRS) can be classified into distinct phenotypes based on the duration and severity of symptoms, the presence of atopy and comorbidities, and the occurrence or absence of nasal polyps. Type 2 inflammation is well characterized in both allergic rhinitis (AR) and CRS with nasal polyps (CRSwNP), whereas type 1 inflammation is predominantly observed in infectious rhinitis and CRS without nasal polyps (CRSsNP). In addition, a neurogenic endotype has been demonstrated in several forms of non-allergic rhinitis. Finally, epithelial barrier dysfunction, documented in AR and CRSwNP, appears to represent an initiating mechanism that may predispose certain patients to the development of type 2 inflammation [42]. Inflammation mechanisms and involved cytokines and cells are summarized in Figure 1.

The treatable traits framework, which identifies and targets specific pathophysiological mechanisms and modifiable factors, is increasingly advocated for the management of patients with overlapping airway diseases [3,5,12,15,33,34].

## 4. Diagnostics Tools

Told of the close link between these pathologies, diagnosis, and subtyping of UAD requires a comprehensive, multidisciplinary approach (Table 1).

Clinical history and physical examination remain crucial, but should be complemented by objective assessments:

*a.*  *Imaging:*

The most used, both in the field of upper and lower airways, are computed tomography (CT) and magnetic resonance imaging (MRI). Crucial for evaluating sinus and lower airway involvement, including detection of parenchymal or structural abnormalities (i.e., emphysema and bronchiectasis) [32].

Moreover, several studies performed in the pediatric setting show that CT combined with accurate clinical indices demonstrated high diagnostic accuracy for OSA (Obstructive Sleep Apnea Syndrome) [43].

Accurate assessment of nasal pathology requires an initial endoscopic examination, which provides direct visualization of the disease and allows for biopsy when indicated. Although nasal endoscopy is indispensable for both diagnosis and clinical monitoring, its utility is limited in evaluating the deeper paranasal sinuses, particularly the frontal, sphenoidal, and posterior ethmoidal regions. Consequently, imaging is necessary to achieve a comprehensive appraisal of disease burden. In patients considered for biologic therapy in the context of severe, uncontrolled chronic rhinosinusitis with nasal polyps (CRSwNP), non-contrast maxillofacial computed tomography (CT) is especially valuable. This modality not only quantifies disease severity through the Lund–Mackay score but also documents the extent of prior surgical intervention using the Amsterdam Classification of Completeness of Endoscopic Sinus Surgery (ACCESS) score—two standardized metrics that support therapeutic indication and establish a baseline for longitudinal evaluation [44,45].

A different and more detailed variant of the Lund-Mackay system is the Zinreich Score, which uses the same anatomical regions but applies a more granular scale, ranging from 0 to 5 for each sinus: 0 = no opacification; 5 = complete opacification. This scoring method offers greater sensitivity in detecting subtle changes over time, making it particularly useful in longitudinal studies or detailed clinical trials. However, due to its complexity, the Zinreich Score is less commonly used in routine clinical practice in favor of the simplest and commonly used Lund MacKay [46].

In the context of the use of biologic therapy, CT must help to exclude alternative diagnoses and detect post-surgical complications such as cerebrospinal fluid (CSF) leaks, reported after FESS. EPOS/EUFOREA 2023, focusing response assessment on endoscopy, symptoms, smell, systemic steroid/salvage surgery needs, and comorbidities, incorporating CT-based measures (e.g., LMS and ACCESS) could provide objective outcome tracking and enhance safety monitoring in severe, uncontrolled type-2 CRSwNP. Emerging radiomics and artificial-intelligence tools may further standardize objective CT evaluation in this setting [44].

Therefore, CT scan remains the better methodology for evaluating nasal anatomy and sinonasal disease, together with fiber-optic rhinoscopy, due to its high spatial resolution and capacity to easily delineate bone structures.

On the other hand, magnetic resonance imaging (MRI), plays an important complementary role in discriminating inflammatory tissue, retained secretions in sinus, and neoplastic lesions, due to its better soft tissue contrast.

An interesting and less common use of imaging techniques, particularly CT, is the interconnection between upper and lower airways severity of diseases. Ethmoidal sinuses and ostiomeatal complexes are more often affected in patients with mild asthma, whereas maxillary, frontal, and sphenoidal sinuses are mostly involved in severe asthma [47].

*b.*  *Allergy and Laboratory Tests:*

Skin prick testing, serum-specific IgE, and peripheral eosinophil counts help define allergic and eosinophilic phenotypes [2,32].

Diagnosis and management of UAD require an integrated approach that includes diagnostic tests to identify different phenotypes, particularly allergic and eosinophilic ones.

Diagnosis of allergies is based on a combined series of clinical, laboratory, and molecular tests.

Skin prick tests and the measurement of specific IgE in the blood remain the most used tests to identify allergic sensitization [48]. Third-level tests use modern technologies (ImmunoCAP, microarray, multiplex test), allowing multiple allergens to be evaluated simultaneously. CRD (Component Resolved Diagnosis) tests identify the specific allergenic molecules responsible for the reaction, distinguishing between true and cross-reactive allergens, improving risk stratification and personalization of therapy Cellular functional tests. The Basophil Activation Test (BAT) and the Mast Cell Activation Test (MAT) are in vitro tests that evaluate the cellular response to allergens, useful for monitoring the effectiveness of therapies or in doubtful cases, but not routinely used to identify a sensitization.

The count of eosinophils, principally in blood but also available in sputum, is a useful biomarker for diagnosis, phenotype detection, and therapeutic choice [49]. Eosinophilic airway diseases present heterogeneous phenotypes such as eosinophilic asthma, nasal polyposis, eosinophilic COPD, ABPA, and EGPA [50,51,52].

The advantages of the methods guarantee, in addition to greater diagnostic accuracy, the possibility of personalizing the therapy with the approach “treatable traits” by identifying and treating specific traits (eosinophilic inflammation, allergic sensitization, etc.) through targeted tests, abandoning the old therapeutic constructs and moving towards more modern precision medicine. The limits linked to complex interpretation, high costs, and the need for standardization of procedures remain, but new horizons are opening, which will include the need for collaboration between allergists, laboratory workers and clinicians.

*c.*  *Pulmonary Function Testing (PFT):*

Spirometry and other PFTs assess lower airway function and obstruction [2,32].

Spirometry remains a pivotal element for the evaluation of lung function in airway pathologies and can be associated with other simple functional tests to better detect small airway dysfunction and early-stage disease (i.e., IOS, FOT).

The combination of PFT and other tests is crucial for the management of the UADs. In a 2019 study, it sought to determine differences in asthma control (ASTHMA Control Questionnaire ACQ-6), lung function (spirometry), and T2 biomarkers (FeNO and Eos) in relation to the presence of allergic rhinitis in patients with allergic asthma: the result was that subjects with allergic asthma and allergic rhinitis showed much lower predicted FEV1% and predicted FEF25-75% than those with asthma alone, indicating worse lung function and significant type 2 inflammation.

Although spirometry is widely used, it is not completely precise in detecting small airway pathology, and the use of normal spirometry may also not be well correlated with the patient’s symptoms. The use of impulse oscillometry (IOS) and forced oscillation technique (FOT) is more sensitive to detect small airway dysfunction and appears to be easy. IOS can detect, better than normal spirometry, the presence of small airway dysfunction in patients with normal spirometry values, but who remained symptomatic [53]. Despite that, the use of both IOS and FOT is to date limited to research centers and is rarely used routinely. Spirometry is also crucial in the management of severe asthmatic patients, both in the timing observation of patients’ lung function, and to evaluate the concept of clinical remission, requiring usually a stabilization of FEV1 or an improvement of more than 80% [54].

*d.*  *Biomarkers:*

Fractional exhaled nitric oxide (FeNO) is a validated, non-invasive biomarker of type 2 inflammation [2,32], reflecting IL-4/IL-13 driven processes in both the upper and lower airways, particularly the activity of IL-13. Elevated FeNO levels are associated with T2 inflammation and, in asthma, can assist in classifying patients into T2-high or T2-low phenotypes. Despite its clinical utility, FeNO alone is insufficient to fully discriminate airway disease phenotypes, especially in severe or complex cases. Indeed, FeNO levels, like eosinophil counts, may be influenced by comorbidities, corticosteroid use, and environmental exposures, and therefore may not reliably identify all patients with low T2 inflammation.

Commonly used biomarkers such as peripheral eosinophilia, total IgE, and exhaled nitric oxide, even when combined, remain suboptimal for distinguishing the diverse phenotypes of type 2 asthma. This limitation is particularly evident in severe asthma, where the challenge of selecting the most appropriate biologic therapy persists.

Sputum inflammatory cell analysis is considered the most specific biomarker for identifying eosinophilic inflammation in asthma and is regarded as the most reliable tool in clinical trials and experimental models. However, its clinical applicability is constrained by several practical limitations. Consequently, there is an increasing need to identify novel biomarkers. Recent studies have highlighted the potential role of microRNAs and nasal methylome signatures as emerging biomarkers in united airway disease, given their involvement in inflammatory pathways. Although the number of studies investigating the role of microRNAs in upper and lower airway inflammatory diseases is growing, only a few have demonstrated a direct connection between these two regions [13].

The nasal cellular epigenome has also been proposed as a biomarker of airway pathology and environmental responsiveness. A 2019 study involving 547 children demonstrated that nasal DNA methylation is associated with asthma, allergy, and airway inflammation. Furthermore, asthma, IgE, and FeNO were linked to accelerated nasal epigenetic aging, underscoring the nasal epigenome as a sensitive biomarker of airway disease [55].

Combining FeNO with additional biomarkers—such as blood eosinophil counts, total IgE, transcriptomic profiles, or epigenetic markers—may enhance diagnostic accuracy, improve phenotyping, and optimize disease monitoring and therapeutic response [56,57].

*e.*  *Histopathology:*

The concept of United Airways Diseases (UADs) is also supported by histopathological and molecular evidence: the nasal and bronchial epithelium constitutes, indeed, a functional and immunological continuum. Damaged epithelium releases cytokines (TSLP, IL-25, IL-33), that activate dendritic cells and trigger the Th2 and ILC2 responses along the rhino-bronchial axis, justifying an integrated diagnostic and therapeutic approach across pulmonology, allergology, and otolaryngology [2,58]. In Th2 high phenotypes, nasal and bronchial tissue show an eosinophilic infiltration, goblet cell hyperplasia with mucus hypersecretion, basement membrane thickening, and signs of epithelial remodeling [59,60]. In contrast, in patients who show bronchiectasis, there is a predominantly neutrophilic inflammation, with epithelial necrosis and destruction of the bronchial wall. In patients with both asthma and bronchiectasis, a mixed eosinophilic-neutrophilic pattern is observed [61].

When the non-invasive biomarkers are inconclusive to detect the underlying inflammatory pattern, tissue biopsy might be performed and may guide the choice between inhaled corticosteroids, antibiotics, or targeted biological therapies [2,32]. The Interasma (Global Asthma Association) recommends the reciprocal evaluation of upper and lower airways in patients with rhinitis or rhinosinusitis to ensure timely diagnosis and integrated management [2,62,63,64]. Other noninvasive tools could be represented by exhaled breath condensate (EBC) and nasal transcriptomics [4,65].

The UAD approach, therefore, requires integrated pathways that maximize therapeutic precision, minimize ineffective treatments, and optimize functional outcomes and quality of life.

## 5. Management Strategies

In pulmonary management, the primary goal should be to ensure good symptom control, reduce inflammation, and prevent exacerbations, with the aim of reducing the need for OCS. According to GINA guidelines, inhaled corticosteroids are the cornerstone therapy and should be administered at the lowest effective dose [66]. Diagnosis and monitoring include spirometry with bronchodilator testing, peak expiratory flow diaries, and bronchial provocation tests when indicated [67]. Another time, GINA guidelines suggest that for patients whose conditions are poorly controlled despite maximum therapy or who are dependent on steroid treatment, the initiation of biological therapy should be considered, chosen based on biomarkers [68,69,70]. To perform an endotipization, GINA suggests using blood eosinophil count (BEC), induced sputum analysis (eosinophilia > 3%) [63,71], and non-invasive tools (FeNO, exhaled breath condensate) to distinguish type 2 inflammation from the non-type 2 forms [62,65].

On the nasal front, EUFOREA suggests that it is essential to optimize topical treatment with topical corticosteroids and nasal washes. When medical therapy is insufficient or anatomy restricts drug delivery, endoscopic sinus surgery is indicated.

A stepwise program is obviously necessary, involving a series of progressively more extensive actions for the management and subsequent follow-up of upper and lower respiratory tract diseases, which also includes the assessment and subsequent therapeutic management of associated comorbidities (GERD, bronchiectasis, AERD) [66,67]. Patient education and adherence to therapy are of crucial relevance, as well as the implementation of a structured follow-up program in which patient-reported outcomes (PROs) are evaluated using appropriate validated tools.

## 6. Multidisciplinary Care

As previously discussed, the management of UAD should be multidisciplinary and involve different experts for a comprehensive assessment of the upper and lower respiratory tract, combining medical, biological and surgical strategies. The aim of these shared evaluation should be the decreasing of the number of exacerbations, the need of OCS and the revision surgery. The guidelines embrace this cross-airway approach: the GINA 2025 update highlights the impact of comorbid rhinosinusitis/nasal polyposis on asthma control and recommends systematic upper-airway evaluation, while EPOS/EUFOREA 2023 delineates indications and response criteria for biologics in CRSwNP and encourages shared biomarker use and joint follow-up [72]. As evidence of this, in patients with CRSwNP and asthma, the medical and surgical outcome of the nasal condition is closely linked to the severity of the bronchial condition: severe asthma is associated with higher polyp recurrence, more revision surgery, and smaller gains in smell and global symptoms after ESS [73]. On the other hand, treating nasal conditions (with topical or biological therapy) improves asthma symptom control and respiratory function [74].

Treat UAD as one disease expressed in two compartments—co-assess, co-manage, and co-monitor—using guideline-aligned, biomarker-informed, and surgery-enabled strategies tailored to endotype and disease burden. Emerging integrative reviews and translational work continue to validate this unified model [27].

## 7. Pharmacotherapy

*a.*  *Intranasal Corticosteroids and associations with antihistamines:*

Intranasal corticosteroids (INCS) are first-line therapy for the treatment of allergic rhinitis and CRSwNP, reducing type-2 inflammation, mucosal edema, polyp size, nasal obstruction, rhinorrhea, and smell impairment, with a positive effect on lower airways [3,6,7]. Administration of these drugs needs to be started early and maintained long term, seeking to optimize administration through patient education and nasal irrigation [3].

Second-generation nasal steroids are highly effective and maintain low systemic bioavailability even with extended use. Variability in response to this treatment can be linked to endotypes: T2 patients respond well, while those without eosinophilic inflammation may need additional therapies [2,3]. 

Advanced delivery systems (steroid-eluting implants, exhalation-delivery sprays) and high-volume, low-pressure steroid irrigation can optimize and improve outcomes, especially on postoperative interventions and polyp regrowth in the immediate healing phase [27,75], with better results in SNOT-22 and endoscopic scores [3]. Even when biologics are introduced, INCS remain the local anti-inflammatory backbone pre- and post-operatively to minimize systemic corticosteroid exposure and maintain disease control over time [1,3,7].

*b.*  *Inhaled Corticosteroids:*

ICS are the cornerstone of asthma treatment, as they are essential for reducing airway inflammation. Treatment should be tailored to the patient using biomarkers and respiratory function as indicators, always maintaining the minimum effective treatment. Regarding the most usable markers, there is FeNO, able to detect bronchial T2 inflammation and to suggest the management of ICS, improving or reducing dose. Furthermore, BEC can be used, even if its count is not correlated with the ICS dose. Inhaled steroid therapy has proven to be highly effective, reducing exacerbations by 50% compared to bronchodilator therapy alone. In addition, therapeutic strategies such as MART (maintenance and reliever therapy) with ICS–formoterol offer rapid anti-inflammatory coverage during symptoms and reduce hospitalizations and the use of oral OCS [76,77]. ICS efficacy depends, also, on practical aspects: verification of inhalation technique, use of spacers in patients not able to use MDI, adherence monitoring, and surveillance for local and systemic effects [77]. More often, ICS are associated with long-acting beta agonists (LABA) to improve bronchodilation. The use of ICS is also effective on the nasal mucosa thanks to systemic diffusion and retrograde mucociliary drainage [2,33]. Moreover, segmental bronchial provocation studies have shown that inflammation induced in the bronchi can extend to the nasal mucosa, highlighting the bidirectionality of the inflammatory process [78].

*c.*  *Antimuscarinics (LAMA):*

In addition to ICS/LABA, in asthmatic patients, the use of LAMA is suggested in GINA and other international guidelines, due to their bronchodilation action and the low anti-inflammatory effect on lower airways [79].

*d.*  *Oral Corticosteroids:*

In the treatment of CRSwNP, OCS are reserved for severe exacerbations or cases that are refractory to therapy, after optimization of the latter. This treatment aims to provide rapid relief from symptoms when needed, while limiting cumulative systemic exposure. Although they are effective in reducing the size of polyps and improving symptom control and quality of life, their effect is temporary and wears off within 3–6 months [3]. OCS represent a fundamental therapeutic option in the treatment of asthma exacerbations, increasing bronchodilation, bronchus oedema, and suppressing inflammation, reducing hospitalization and the risk of recurrence [80]. Considering the short- and long-term risks of using these drugs, all the guidelines both in asthma and CRSwNP suggest a steroid-sparing approach, with biologics [3]. Therefore, OCS should be limited to short, carefully timed courses, with shared decision-making and longitudinal monitoring of cumulative exposure.

*e.*  *Antibiotics:*

Indicated for acute bacterial exacerbations or chronic infection, particularly in bronchiectasis [9,33]. Up to 85% of sinusitis cases resolve spontaneously within 7–15 days, without the need for antibiotic therapy. This therapy should therefore be reserved for severe cases, with symptoms persisting for more than 10 days, and for patients who show a worsening of symptoms after an initial improvement. According to the 2025 American Academy of Otolaryngology–Head and Neck Surgery (AAO-HNSF) guidelines, first-line antibiotics are amoxicillin-clavulanate for 5–10 days, followed by doxycycline or fluoroquinolones for those who are hypersensitive to penicillin. However, the use of macrolides is not recommended due to the high resistance rate of *S. pneumoniae* [81,82,83].

In patients with COPD, asthma, and bronchiectasis, the choice of antibiotic therapy should be based on clinical and microbiological evidence to avoid resistance and microbial alterations. In chronic or treatment-resistant forms, endoscopy with targeted cultures can enable the selection of a more precise and effective treatment [15,84,85,86].

In patients with bronchiectasis, chronic infection fuels a cycle of inflammation, colonization, and structural damage, resulting in clinical manifestations. Generally, in the event of an exacerbation, amoxicillin/clavulanate is again the first choice. Infection with Pseudomonas aeruginosa has a poor prognosis and, when detected for the first time, requires eradication with systemic and inhaled therapy (mainly fluoroquinolones), while in cases of chronic colonization, inhaled antibiotic therapy (tobramycin, colistin) is required. In cases where non-tuberculous mycobacteria are isolated, long-term treatment (12 months after sputum negativity) is indicated and requires targeted antibiotic therapy (macrolide + rifampicin + ethambutol) [15,87,88].

In patients with bronchiectasis and sinonasal comorbidities, integrated infection management may improve both respiratory and ENT outcomes. In asthma, the use of a long period (3 months) of 3 times a week of macrolides could be considered in uncontrolled patients.

*f.*  *Biologic Therapies:*

The introduction of biologics (Omalizumab, Mepolizumab, Benralizumab, Dupilumab and Tezepelumab) has decisively changed the management of patients with severe asthma, significantly reducing exacerbations and the use of OCS, and improving respiratory function and quality of life, allowing for a personalized approach based on biomarkers (FeNO, eosinophil count, IgE). Biological therapy acts on key cytokines (IL-5, IL-5R, IL-4R, TSLP) that orchestrate the inflammatory response and act on both the upper and lower respiratory tract [12,33]. Recent studies have confirmed that the efficacy of these biologics extends to the upper airways: Benralizumab and Mepolizumab have demonstrated significant reductions in nasal inflammation and SNOT 22 scores in patients with comorbid CRSwNP [5,12].

For treatment of CRSwNP, we should follow the international guidelines European Position Paper on Rhinosinusitis and Nasal Polyps (EPOS) and European Forum for Research and Education in Allergy and Airway diseases (EUFOREA), following the strict conditions for prescription [89] and keep the patients in a strict follow-up during the first Targeted biologics have demonstrated efficacy in both upper and lower airway disease, reducing exacerbations, improving control, and decreasing the need for systemic corticosteroids and surgery. This has been demonstrated during phase III trials [90,91], but also during real-life studies [92]. Reviews emphasize that blockade of IL-4/IL-13 and IL-5 pathways not only improves sinonasal outcomes—such as nasal polyp score, congestion, and olfactory function—but also exerts parallel benefits on comorbid asthma, reinforcing the unified airway concept, reducing the use of OCS and the need for EES [93].

*g.*  *Allergen-Specific Immunotherapy:*

Specific immunotherapy (AIT) is a targeted therapeutic strategy for patients suffering from allergic rhinitis, chronic rhinosinusitis, and bronchial asthma, associated with documented allergic sensitization and persistent symptoms despite drug therapy. By modulating type 2 inflammation along the nasobronchial axis, AIT improves the symptoms of rhinitis and asthma, reduces bronchial hyperresponsiveness, the use of drugs, and the number of exacerbations, with benefits in terms of quality of life and airway remodeling [94,95].

Allergen-specific immunotherapy has also been shown to prevent the onset of bronchial asthma and is currently the only therapy that modifies the natural course of the disease. International guidelines also recommend its use in patients with documented sensitization to the allergen, with specific IgE findings, clinically relevant symptoms, and a suboptimal response to conventional therapy, after exclusion of contraindications [2,24,96].

*h.*  *New Targeted Therapies:*

In the context of United Airways Diseases (UADs), there is growing interest in other specific therapies for patients who do not respond adequately to conventional treatments. Among these, JAK inhibitors and synthetic peptides represent new therapies under study. They can reduce inflammation and immune dysregulation along the nasobronchial axis.

Because JAK inhibitors block signaling through the IL-4, IL-5, IL-13, and TSLP cytokines by inhibiting the JAK-STAT pathway, these drugs seem to be able to reduce activation of eosinophils, mast cells, and effector T cells, inhibiting the JAK-STAT pathway, essential for activating the signaling of IL-4, IL-5, IL-13, and TSLP cytokines. Agents such as tofacitinib, baricitinib, and ruxolitinib are currently under investigation for severe eosinophilic asthma and chronic rhinosinusitis with nasal polyposis, till now with promising results in reducing inflammation, improving lung function, and alleviating nasal symptoms, especially in patients with mixed phenotypes or systemic comorbidities, but necessitating further study to be considered as a new possibility for these diseases [34].

Synthetic peptides and peptidomimetics are emerging as innovative therapies interfering with protein–protein interactions, driving immune activation and mucosal inflammation. Among the experimental candidates, antagonists of CXCL10 (eldelumab), anti-TSLP peptides, and mimetics of IL-13Rα1 have demonstrated, in preclinical studies, the ability to reduce eosinophilic infiltrate, mucus secretion, and bronchial hyperreactivity, suggesting benefits for both upper and lower airways [34].

*i.*  *Surgical Interventions:*

It should certainly be emphasized that surgery should be considered in patients who do not respond to topical therapies or other drugs, such as biologics. ESS is indicated for those patients with CRSwNP who are uncontrolled despite optimized medical therapy, including INCS, short courses of oral corticosteroids, and saline irrigation. The principal goals of ESS are to restore sinus ventilation and drainage and to improve the delivery of topical medications, particularly postoperative INCS, which remains the standard of care [3,7]. Beyond an improvement in nasal symptoms, ESS, when necessary and not substitutable by other approaches, has been shown to improve lower airway outcomes with concomitant asthma, thus supporting the concept of UAD. Another time, if not treatable with other therapies and necessitating surgery, patients with eosinophilic CRSwNP and asthma demonstrated a significant reduction in type 2 inflammatory markers after ESS, as well as blood eosinophils and FeNO levels decreased markedly, while lung function (FEV_1_) and asthma control showed improvements [97]. Higher pre-operative tissue eosinophilia and serum periostin have been associated with larger postoperative gains and fewer exacerbations, suggesting a role for biomarkers in prognostication and shared decision-making [98,99].

However, surgical outcomes vary widely between individuals, and polyp recurrence is still frequent, especially in patients with type 2 disease and those with severe asthma. This shows that surgery is just one part of long-term, multidisciplinary management and not a definitive cure [6,7]. When it comes to the extent of surgery, there is no universal consensus on the best approach for chronic rhinosinusitis with nasal polyps (CRSwNP) within the united airway disease framework. Surgical options range from limited and targeted procedures (such as uncinectomy with middle meatal antrostomy, with or without a limited ethmoidectomy), up to complete endoscopic sinus surgery (involving the opening of all paranasal sinus ostia and full ethmoidectomy). The decision depends on factors such as polyp burden, individual anatomy, previous surgeries, and the presence of asthma. However, few studies directly compare limited and complete surgical approaches in terms of lower airway outcomes, so practices can vary significantly between centers [99].

If a patient has extensive nasal polyps and all the paranasal sinuses are blocked, we define a complete ESS as opening all the sinus passages—this includes making larger openings in the maxillary, ethmoid (both front and back), frontal, and sphenoid sinuses. The goal is to help restore normal airflow, improve the natural cleaning process, and make it easier for topical treatments to reach all areas. We also often remove part of the middle turbinate to keep the middle nasal passage clear, make aftercare easier, and lower the risk of scar tissue forming [100,101].

The introduction of biologic drugs (targeting IL-4Rα, IL-5/IL-5R, or IgE) has changed the way clinics decide on treatment. Clinical studies show that, for patients already using nasal steroid sprays, biologics can significantly reduce the need for initial or repeat sinus surgery. They also help shrink polyps, clear up sinus blockages, improve a patient’s sense of smell, and boost overall quality of life. For patients with asthma, these benefits extend to better asthma control. In practice, possible treatment approaches include: (i) starting with biologics in patients who are likely to have a recurrence or have severe asthma alongside their sinus disease; (ii) starting with surgery first if the polyps are so extensive that they block access for topical treatments, then following up with regular nasal steroid sprays and considering biologics if symptoms persist. No matter the path, a common goal is to avoid giving patients too many courses of systemic steroids, which can have serious side effects [1,3,7].

*j.*  *Non-Pharmacologic Measures:*

In the management of chronic rhinosinusitis type 2 with nasal polyposis, several non-pharmacological strategies play a relevant role as important adjuncts for long-term disease control. One of the best-established is nasal saline irrigation. International guidelines [89] recommend its regular use because of its low risk profile, low cost, and ability to improve obstructive symptoms and mucociliary clearance [3].

A Cochrane review confirmed a symptomatic benefit in CRS patients treated with saline irrigation, particularly when delivered as high-volume, low-pressure solutions. Such methods are more effective than standard sprays, including in post-surgical contexts, where they contribute to better endoscopic and radiological outcomes at follow-up [3]. In their trial, Harvey et al., such as Chakapan Promsopa et al., demonstrated that topical intranasal corticosteroids achieve better results in controlling symptoms in post-surgical patients [102] and in non-surgical patients [103].

Other behavioral measures include allergen avoidance and smoking cessation. Continuous exposure to aeroallergens can amplify type 2 inflammation, while cigarette smoke worsens respiratory function and accelerates FEV1 decline. It is important to receive correct nasal irrigation also to get allergens away from the nose and, in this way, lower the type 2-driven inflammation, lowering asthma symptoms also [3].

Another critical aspect is patient education. Usually, it is important to show the patients the exact method they should use when using nasal irrigation and topical intranasal sprays: if not well explained, if not performed correctly, patients may not benefit from local therapy or may even lose adherence to treatment completely.

## 8. Precision Medicine and Treatable Traits

The treatable traits (TT) paradigm advocates for the identification and targeted management of specific, modifiable disease mechanisms and risk factors, rather than a syndromic approach. The concept of TT currently represents an emerging paradigm for the management of airway diseases that goes beyond the definition of a single disease, but allows the identification of clinical, behavioral, and pathophysiological aspects on which to act with targeted therapeutic interventions. This approach allows for a multimodal assessment of bronchial asthma, but also its main comorbidities, such as AR, CRS, and CRSwNP, fitting fully within the concept of UADs [104,105].

## 9. Research Gaps and Future Directions

Despite advances, significant gaps remain in understanding and managing UAD. There is a paucity of high-quality studies specifically addressing integrated treatment strategies, optimal biomarker use, and long-term outcomes in patients with overlapping airway diseases. Future research priorities include [11,34] elucidation of the molecular and microbial mechanisms linking upper and lower airway inflammation [13,27,34] development and validation of biomarkers for diagnosis, phenotyping, and monitoring response to therapy [11,32,34], evaluation of novel therapeutics, including JAK inhibitors and synthetic peptides, in refractory UAD [34] and implementation of multidisciplinary care models and real-world studies to assess the impact of integrated management on patient outcomes [2,4,11]. Future research should prioritize three key areas. First, large-scale prospective studies could be useful to establish standardized protocols and evaluate the long-term effectiveness of unified-airway interventions across diverse patient populations. Second, mechanistic investigations into the shared inflammatory pathways and molecular drivers of airway disease could yield novel therapeutic targets and biomarkers for precision medicine. Third, the role of emerging technologies—such as digital health platforms, artificial intelligence, and minimally invasive diagnostics—should be explored to enhance early detection and personalized care. By addressing these priorities, the field can move toward a more holistic and evidence-based framework that advances both clinical practice and patient well-being.

## Figures and Tables

**Table 1:** Main diagnostic tools for UAD.

Category	Main Tools	Objectives/Utility	Limitations
**Imaging**	CT, MRI, nasal endoscopy	Evaluation of anatomy and CRS severity; Lund–Mackay and ACCESS scores; exclusion of complications	CT: radiation exposure; MRI: cost and availability; endoscopy limited to deeper sinuses
**Allergy and Laboratory Tests**	Skin prick test, serum IgE, peripheral eosinophil count, CRD, BAT/MAT	Identification of allergic sensitization; eosinophilic/allergic phenotyping	Complex interpretation; high cost; lack of standardization
**Pulmonary Function**	Spirometry, Impulse Oscillometry (IOS), Forced Oscillation Technique (FOT)	Assessment of lower airway function; detection of small airway dysfunction; asthma monitoring	Spirometry less sensitive for small airways; IOS/FOT rarely used in routine practice
**Biomarkers**	FeNO, blood eosinophils, IgE, microRNAs, nasal epigenome	Differentiation of T2-high vs. T2-low phenotypes; monitoring therapeutic response	Influenced by corticosteroids/environment; emerging biomarkers not yet validated
**Histopathology**	Nasal or bronchial biopsy	Confirmation of inflammatory pattern (eosinophilic/neutrophilic); guidance for therapy	More invasive; not usually performed (bronchial)

## Data Availability

No new data were created or analyzed in this study. Data sharing is not applicable.

## Abbreviations

The following abbreviations are used in this manuscript:

ABPA	Allergic Bronchopulmonary Aspergillosis
ACCESS	Amsterdam Classification of Completeness of Endoscopic Sinus Surgery
ACQ-6	Asthma Control Questionnaire-6
AERD	Aspirin-Exacerbated Respiratory Disease
AIT	Allergen-Specific Immunotherapy
AR	Allergic Rhinitis
BAT	Basophil Activation Test
COPD	Chronic Obstructive Pulmonary Disease
CRD	Component Resolved Diagnosis
CRSsNP	Chronic Rhinosinusitis without Nasal Polyps
CRSwNP	Chronic Rhinosinusitis with Nasal Polyps
CSF	Cerebrospinal Fluid
CT	Computed Tomography
EBC	Exhaled Breath Condensate
EGPA	Eosinophilic Granulomatosis with Polyangiitis
Eos	Eosinophils
EPOS/EUFOREA	European Position Paper on Rhinosinusitis and Nasal Polyps/European Forum for Research and Education in Allergy and Airway Diseases
ESS	Endoscopic Sinus Surgery
FEF25-75%	Forced Expiratory Flow at 25–75% of Vital Capacity
FeNO	Fractional exhaled Nitric Oxide
FEV1%	Forced Expiratory Volume in 1 s (percentuale del predetto)
GERD	Gastroesophageal Reflux Disease
GINA	Global Initiative for Asthma
ICS	Inhaled Corticosteroids
IgE	Immunoglobulins E
IL-4, IL-5, IL-13	Interleukin-4, Interleukin-5, Interleukin-13 (citokines)
IL-4R	Interleukin-4 Receptor
IL-4Rα	Interleukin-4 Receptor alpha
IL-5R	Interleukin-5 Receptor
ILC2s	Type 2 Innate Lymphoid Cells
INCS	Intranasal Corticosteroids
IOS	Impulse Oscillometry (o Spirometria con Tecnica di Oscillazione)
JAK	Janus Kinase (inibitori)
LABA	Long-Acting Beta Agonist
LAMA	Long-Acting Muscarinic Antagonist
LMS	Lund-Mackay Score
MART	Maintenance and Reliever Therapy
MAT	Mast Cell Activation Test
MDI	Metered-Dose Inhaler
MRI	Magnetic Resonance Imaging
OCS	Oral Corticosteroids
OSA	Obstructive Sleep Apnea Syndrome
PFT	Pulmonary Function Testing
PROs	Patient-Reported Outcomes
SNOT-22	Sino-Nasal Outcome Test-22 (score)
STAT	Signal Transducer and Activator of Transcription
T2	Type 2 (Inflammation)
Th2	T helper type 2 (cells)
TSLP	Thymic Stromal Lymphopoietin (citokine)
TT	Treatable Traits
UAD	United Airway Disease
